# Associations between Circulating VEGFR2^hi^-Neutrophils and Carotid Plaque Burden in Patients Aged 40-64 without Established Atherosclerotic Cardiovascular Disease

**DOI:** 10.1155/2022/1539935

**Published:** 2022-04-26

**Authors:** Vadim Genkel, Ilya Dolgushin, Irina Baturina, Albina Savochkina, Karina Nikushkina, Anna Minasova, Alla Kuznetsova, Igor Shaposhnik

**Affiliations:** ^1^Department of Internal Medicine, Federal State Budgetary Educational Institution of Higher Education “South-Ural State Medical University” of the Ministry of Healthcare of the Russian Federation, 454092 Chelyabinsk, Russia; ^2^Department of Microbiology, Virology, Immunology and Laboratory Medicine, Federal State Budgetary Educational Institution of Higher Education “South-Ural State Medical University” of the Ministry of Healthcare of the Russian Federation, 454092 Chelyabinsk, Russia; ^3^Research Institute of Immunology, Federal State Budgetary Educational Institution of Higher Education “South-Ural State Medical University” of the Ministry of Healthcare of the Russian Federation, 454092 Chelyabinsk, Russia; ^4^Department of Hospital Therapy, Federal State Budgetary Educational Institution of Higher Education “South-Ural State Medical University” of the Ministry of Healthcare of the Russian Federation, 454092 Chelyabinsk, Russia

## Abstract

**Background:**

Neutrophils expressing vascular endothelial growth factor receptor (VEGFR) represent a distinct subtype of neutrophils with proangiogenic properties. The purpose of this study was to identify the interrelations between circulating CD16^hi^CD11b^hi^CD62L^lo^CXCR2^hi^VEGFR2^hi^-neutrophils and indicators of carotid plaque burden in patients without atherosclerotic cardiovascular diseases (ASCVD).

**Methods:**

The study included 145 patients, 51.7% men and 48.3% women, median age—49.0 years. All patients underwent carotid duplex ultrasound scanning. The maximal carotid plaque thickness was used as an indicator of carotid plaque burden. Also, carotid intima-media thickness (cIMT) and femoral IMT were measured. The phenotyping of neutrophil subpopulations was executed by the flow cytometry via the Navios 6/2*. Results*. The subpopulation of VEGFR2^hi^-neutrophils accounted for about 5% of the total pool of circulating neutrophils. A decrease in VEGFR2^hi^-neutrophils with an increase in carotid plaque burden was statistically significant (*p* = 0.036). A decrease in VEGFR2^hi^-neutrophils < 4.52% allowed to predict the presence of plaque with a maximum height > 2.1 mm (Q4), with sensitivity of 78.9% and specificity of 61.5% (AUC 0.693; 95% CI 0.575-0.811; *p* = 0.007). Inverse correlations were established between the carotid and femoral IMT and the absolute and relative number of VEGFR2^hi^-neutrophils (*p* < 0.01).

**Conclusion:**

In patients aged 40-64 years without established ASCVD, with an increase in indicators of the carotid plaque burden, a significant decrease in the proportion of circulating VEGFR2^hi^-neutrophils was noticed. A decrease in the relative number of VEGFR2^hi^-neutrophils of less than 4.52% made it possible to predict the presence of extent carotid atherosclerosis with sensitivity of 78.9% and specificity of 61.5%.

## 1. Introduction

The study of the role of neutrophils within the process of the initiation and progression of atherosclerosis and the development of complications have only been actively conducted over the past few years [1]. This is largely due to new discoveries in the field of neutrophil biology, which has made it possible to revise traditional data concerning the duration of their lives and the homogeneity of the composite population [2]. The discovery of new pathophysiological mechanisms through which neutrophilic granulocytes affect atherogenesis and the development of cardiovascular diseases (CVD) has led to their consideration as therapeutic targets and has given rise to a variety of clinical studies [3, 4].

Massena et al. identified a new subpopulation of circulating neutrophils expressing vascular endothelial growth factor receptor (VEGFR1, CD49d^+^VEGFR1^high^CXCR4^high^-neutrophils) and constituting about 5% of the total pool of circulating neutrophils [5]. It was found that neutrophils of this subtype are able to be recruited into the ischemic tissues due to the fact that VEGF-A secreted in the ischemic tissue induces VEGFR-stimulated chemokinesis in neutrophils [5, 6]. In the VEGFR^high^ ischemic tissue, neutrophils realize their proangiogenic effects in several known ways: secretion of VEGF-A contained in neutrophil granules, secretion of growth factor Bv8, and release of matrix metalloproteinase 9 (MMP-9), which provides for the remodeling of the extracellular matrix (ECM) and the activation of proangiogenic factors [7–9]. Today, research on angiogenic neutrophils is conducted mainly within the framework of studies of tumor-associated neutrophils and their role in the development of resistance to anti-VEGF therapy [10]. Moreover, it can be assumed that VEGFR^high^-neutrophils are integral in the pathogenesis of chronic inflammatory diseases and atherosclerosis [8].

In experimental and clinical studies, it has been demonstrated that extensive hypoxia is found in the tissues of atherosclerotic plaque (AP) [11]. Moreover, even in the early stages of arterial remodeling, manifested by an increase in vascular stiffness and hyperplasia of intima and media, local hypoxia develops, presumably associated with vasa vasorum (VV) compression [12, 13]. In turn, hypoxia induces an increase in the local expression of hypoxia-inducible factor 1*α* (HIF-1*α*), followed by the synthesis of the proangiogenic (VEGF) and proinflammatory factors [14, 15]. The level of the local expression of HIF-1*α* strongly correlates with the number of VEGFR, CD34, and CD133-positive cells recruited into the atheroma [14]. VEGFR^high^-neutrophils mobilized into the vascular wall from the systemic circulation secrete VEGF, MMP-9, and neutrophil gelatinase-associated lipocalin (NGAL), which stimulates neovascularization of AP, ECM degradation with subsequent growth, and the destabilization of atheroma [15, 16]. Assessments of circulating VEGFR^high^-neutrophils in patients with subclinical atherosclerosis and atherosclerotic cardiovascular diseases (ASCVD) can serve to provide us with additional diagnostic and prognostic information. The purpose of this study was to study the interrelations between the number of circulating VEGFR2^hi^-neutrophils and indicators of carotid plaque burden in patients without diagnosed CVD.

## 2. Materials and Methods

The study included asymptomatic patients aged 40-64 years with CVD risk factors without established ASCVD. After being enrolled in the study, all patients signed an informed consent form. The study protocol was approved by the Ethics Committee of South-Ural State Medical University (Protocol No. 10 of 27.10.2018). The study exclusion criteria and/or study drop-out parameters included the presence of the following clinical conditions: the previously diagnosed ASCVD (history of cerebrovascular disease, coronary artery disease, peripheral arterial disease, and revascularization of coronary or peripheral arteries), severe dysfunction of the liver and kidneys (decreased estimated glomerular filtration rate (eGFR) less than 30 mL/min/1.73 m^2^), malignant neoplasms, diagnosed chronic inflammatory diseases (CID), and/or the presence of acute inflammatory or infectious diseases during the previous 28 days.

### 2.1. Duplex Ultrasound Scanning

All patients underwent duplex ultrasound scanning (DUS) of the carotid arteries. The following vessels were examined from both sides in longitudinal and cross-sections along the entire length: common carotid arteries (CCA) with CCA bifurcation, internal carotid arteries (ICA), and external carotid arteries (ECA) from the anterior, lateral, and posterior approaches. The study was executed in B-mode, color doppler mode, and pulsed and power doppler ultrasonography. The study was performed with a linear transducer with a frequency of 10 MHz via the Samsung Medison EKO7 Ultrasound Scanner (Republic of Korea).

The carotid intima-media thickness (cIMT) was determined automatically (AutoIMT function) on both sides in the distal third of the СCA 1 cm proximal to the СCA bifurcation from the anterior approach [17]. The mean cIMT (cIMTm) was determined by the formula  cIMTm = (cIMT CCAleft + cIMT CCAright)/2. IMT was measured in the same way in the common femoral arteries. AP was determined as focal thickening of the IMT more than 1.5 mm or 0.5 mm larger than the surrounding IMT or 50% larger than the IMT of the adjacent areas [17]. The percentage of stenosis was measured planimetrically in B-mode over the diameter in the cross-section of the vessel. The percentage of stenosis was determined according to the ECST method (The European Carotid Surgery Trial) [18]. In the case of detection of AP, stenosing the lumen of the vessels, the maximum percentage of stenosis in a particular patient was determined. The maximal carotid plaque thickness (cPTm) was used as an indicator of carotid plaque burden [19]. The measurements were conducted according to recommendations presented by the American Society of Echocardiography [20].

### 2.2. Laboratory Examination

All patients underwent the complete blood test via an automatic analyzer (Medonic M16, Sweden), for which venous blood was collected into test tubes with anticoagulant K2EDTA.

With at least 8 hours of fasting, the following biochemical laboratory parameters of blood were determined: total cholesterol (TC), low-density lipoprotein cholesterol (LDL-C), high-density lipoprotein cholesterol (HDL-C), triglycerides (TG), glycated hemoglobin, and creatinine with subsequent calculation of estimated glomerular filtration rate (eGFR) according to the CKD-EPI formula (BioChem Analette, USA).

The concentration of high-sensitivity C-reactive protein (hsCRP) and VEGF in blood serum was measured using enzyme-linked immunosorbent assay kits (Vector-Best, Russia).

**Figure 1:**
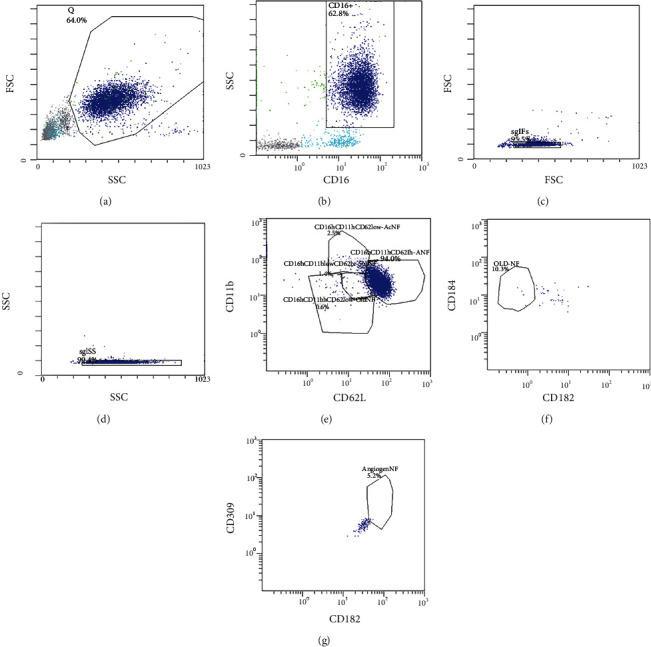
Gating strategy of flow cytometry. Sequential gating strategy for the identification of neutrophil subpopulations. Granulocytes were gated based on Forward Scatter (FSC) and Side Scatter (SSC) (а). Furthermore, CD16^+^ cells were identified, and single cells were gated based on FSC Time-Of-Flight and FSC Integral (c) and SSC Time-Of-Flight and SSC Integral (d). Identification of mature neutrophil subpopulations depending on CD11b and CD62L expression (CD11b^hi^CD62L^hi^) (e). Identification of aging neutrophils by the expression of CD184 and CD182 (f). Identification of VEGFR2^hi^-neutrophils by the expression of CD309 and CD182 (g).

The phenotyping and differentiation of neutrophil subpopulations were executed by the flow cytometry via the Navios 6/2 (Beckman Coulter, USA). With at least 8 hours of fasting, blood sampling was routed into test tubes containing the anticoagulant K2EDTA. The antibody drug conjugates were used for phenotyping and differentiation of neutrophil subpopulations: CD16, PE-Cyanine7 (Invitrogen, USA); CD11b-FITC (Beckman Coulter, USA); CD62L-PE (Beckman Coulter, USA); CD184 (CXCR4)-PE-CF594 (BD Biosciences, USA); CD182 (CXCR2)-PerCP-eFluor 710 (BD Biosciences, USA); and CD309 (VEGFR2)–Alexa Fluor 647 (BioLegend, USA). The gating strategy of flow cytometry is represented in Figure 1.

### 2.3. Statistical Analysis

The analysis of the obtained data was performed with the help of the IBM SPSS Statistics and Statistical Data Analysis Package, Version 18, MedCalc, Version 20.019. Qualitative variables were described by absolute and relative frequencies (percentages). Quantitative variables were described with the following statistics: median (Me) and 25th and 75th percentiles (LQ, UQ) in the case of nonnormal distributed variables. In order to determine the interrelations of indicators, Spearman correlation analysis was used.

Kruskal-Wallis rank-based variance analysis was calculated with subsequent a posteriori calculation of the Mann–Whitney test to assess the significance of differences between more than two groups. Differences were considered statistically significant at the critical significance level of 0.05. To assess the dependence of one quantitative variable on another, the linear regression procedure was executed. To assess the dependence of one qualitative variable on another, the logistic regression procedure was performed. To establish the threshold values of the studied parameters, ROC analysis was performed with the determination of sensitivity and specificity, as well as the calculation of the area under the characteristic curve (AUC) with a 95% confidence interval (CI).

## 3. Results

The study included 145 patients, 75 (51.7%) men and 70 (48.3%) women; the median age was 49.0 years. Detailed characteristics of the subjects are presented in Table 1.

### 3.1. Subpopulation Composition of Neutrophils in the Test Group of Patients and the Interrelations of the Number of VEGFR2^hi^-Neutrophils with Clinical and Laboratory Characteristics

Table 2 demonstrates the results of the performed study of the subpopulation composition of neutrophils in accordance with flow cytometry data.

As can be seen from the data presented in Table 2, the subpopulation of VEGFR2^hi^-neutrophils accounted for about 5% of the total pool of circulating neutrophils, which is consistent with the previously published data [5].

According to the Spearman correlation analysis, the absolute and relative number of proangiogenic neutrophils did not correlate with the demographic, clinical, or biochemical parameters assessed in the study, including the serum VEGF level.

It is also important to note that the absolute (173.5 cells/*μ*l (83.5; 289.0) vs. 137.0 cells/*μ*l (92.5; 236.7); *p* = 0.924) and relative (5.19% (2.46; 8.17) vs. 4.76% (2.84; 7.23); *p* = 0.724) VEGFR2^hi^-neutrophil counts were not significantly different in patients with AP compared with patients without plaques.

### 3.2. Interrelations of the Number of VEGFR2^hi^-Neutrophils with Indicators of Carotid Plaque Burden

**Figure 2:**
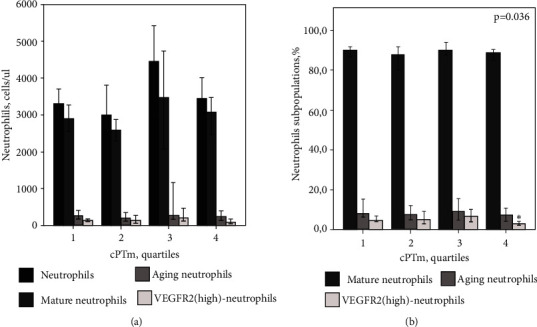
Absolute (a) and relative (b) neutrophil count as a function of cPTm quartile.

Patients were divided into quartiles depending on cPTm values. The following quartiles (*Q*) are defined for the cPTm values: Q1 ≤ 1.40 mm, Q2 = 1.40-1.70 mm, Q3 = 1.71-2.10 mm, and Q4 ≥ 2.10 mm. Features of the quantitative composition of the test subpopulations of neutrophils based on the cPTm values are presented in Table S1 and Figure 2.

It should be noticed that the absolute number of VEGFR2^hi^-neutrophils did not significantly change from Q1 to Q2. However, there was a tendency to a decrease in the number of cells of this subtype with an increase in cPTm (*p* = 0.058). In the percentage ratio, a decrease in VEGFR2^hi^-neutrophils with an increase in carotid plaque burden was statistically significant (*p* = 0.036). In the pairwise comparison using the adjusted Mann–Whitney test, the content of VEGFR2^hi^-neutrophils in patients from Q4 was significantly lower in comparison with Q3 (3.08% (2.04; 4.91) versus 6.66% (3.40; 8.84); *p* = 0.026).

**Figure 3:**
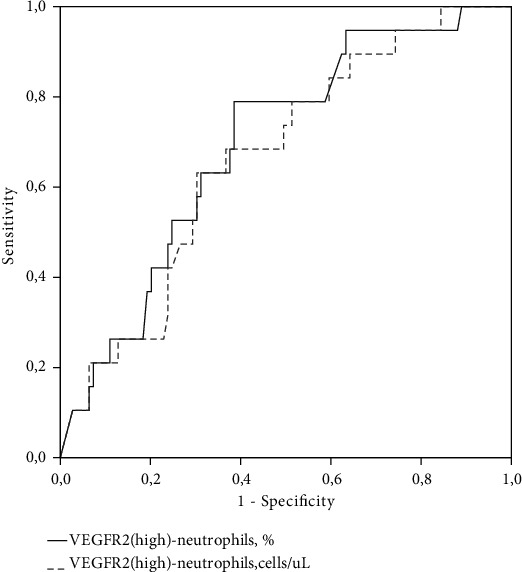
ROC curve demonstrating the diagnostic significance of VEGFR2^hi^-neutrophils in relation to the high carotid plaque burden (cPTm > 2.1 mm (Q4)).

To determine the diagnostic value of VEGFR2^hi^-neutrophils in relation to the presence of high carotid plaque burden, ROC analysis was executed (see Figure 3).

A decrease in the relative number of VEGFR2^hi^-neutrophils of less than 4.52% allowed us to predict the presence of AP with a maximum height > 2.1 mm, with sensitivity of 78.9% and specificity of 61.5% (AUC 0.693; 95% CI 0.575-0.811; *p* = 0.007). A decrease in the absolute number of VEGFR2^hi^-neutrophils less than 128 cells/*μ*l made it possible to predict the presence of AP with a maximum height > 2.1 mm, with sensitivity of 68.4% and specificity of 63.3% (AUC 0.666; 95% CI 0.544-0.787; *p* = 0.022). According to logistic regression analysis, a decrease in the relative number of VEGFR2^hi^-neutrophils of less than 4.52% was associated with the odds ratio (OR) of the presence of cPTm corresponding to Q4 7.39 (95% CI 1.05-52.0; *p* = 0.045) after adjusting for gender, age, hypertension, diabetes mellitus, obesity, smoking, LDL-C, eGFR, hsCRP, intake of beta-blockers, renin-angiotensin system inhibitors, and statins.

### 3.3. Interrelations between the Number of VEGFR2^hi^-Neutrophils and Carotid and Femoral IMT

**Figure 4:**
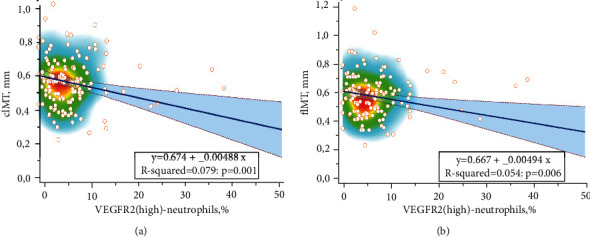
Relationships of VEGFR2^hi^-neutrophils with carotid (a) and femoral IMT (b).

According to the correlation analysis, inverse correlations were established between the cIMTm and the absolute (*r* = −0.270; *p* = 0.002) and relative (*r* = −0.281; *p* = 0.001) number of VEGFR2^hi^-neutrophils. Femoral IMTm also was inversely correlated with the absolute (*r* = −0.241; *p* = 0.006) and relative (*r* = −0.233; *p* = 0.006) number of VEGFR2^hi^-neutrophils. Linear regression analysis was performed to make the quantitative evaluation of the interrelations between carotid and femoral IMTm and the relative number of VEGFR2^hi-^neutrophils (see Table S2, Figure 4).

Thus, statistically significant interrelations were found between the level of circulating VEGFR2^hi^-neutrophils and carotid and femoral IMTm. At the same time, changes in the relative content of VEGFR2^hi^-neutrophils could explain up to 7.9% (according to the representations of *R*^2^ in a convenient 0-100% scale) of the variability of the IMTm of these arteries.

## 4. Discussion

In the last few years, the accumulation of neutrophils in the AP has been established both by the results of studies with neutrophil-specific fluorescent labeling and by the results of morphological studies [21, 22]. Neutrophils recruited into atheroma take part in the destabilization of the plaque. Silvestre-Roig et al. found that the number of neutrophils in AP inversely correlates with the number of smooth muscle cells and the thickness of the plaque fibrous cap and directly correlates with the volume of the lipid-necrotic core [23]. The attraction of neutrophils from the systemic circulation to the vascular wall is regulated by a large number of chemokines, among which the key ones are CXCR2 ligands—CXCL1, CXCL2, CXCL3, CXCL5, CXCL6, and CXCL8 [24]. In addition, there is a specific subpopulation of circulating neutrophils whose recruitment into the tissue is triggered by local hypoxia through VEGF-dependent chemokinesis [25]. These neutrophils can play an independent role in the growth and destabilization of AP.

The main results of the study are as follows: (1) in patients aged 40-64 years without established ASCVD, as carotid plaque burden increased, there was a significant decrease in the proportion of circulating VEGFR2^hi^-neutrophils; (2) a decrease in the relative number of VEGFR2^hi^-neutrophils less than 4.52% made it possible to predict the presence of extent carotid atherosclerosis (indicators of burden corresponding to Q4) with sensitivity of 78.9% and specificity of 61.5% and was associated with an OR of 7.39 (95% CI 1.05-52.0; *p* = 0.045) after adjusting for confounding factors; and (3) the number of VEGFR2^hi^-neutrophils correlated inversely with carotid and femoral IMT. To our knowledge, this is the first clinical study devoted to the study of the interrelations between proangiogenic neutrophils and subclinical atherosclerosis. The previously established functional features of proangiogenic neutrophils also indicate their possible independent and important role in the progression and destabilization of AP. Thus, neutrophils of this subpopulation are distinguished by a higher ability to destroy the basement membrane and secrete TIMP-free MMP-9, which may contribute to the neovascularization of atheroma and degradation of its fibrous component [25, 26]. Additionally, this can lead to the development of intraplaque hemorrhage, which in turn is associated with an increased risk of atherothrombotic events and a rapid increase in the carotid plaque burden and degree of vascular stenosis [27].

In the present study, a decrease in the relative and absolute number of VEGFR2^hi^-neutrophils was associated with high values of carotid plaque burden and IMT. We think that this could be because in conditions of extent atherosclerosis and systemic vascular remodeling, there can be an increased migration of VEGFR2^hi^-neutrophils into the tissue (mediated by both VEGFR and CXCR2), which leads to a decrease in their number in the systemic circulation [28]. In our opinion, the lack of interrelations between the serum VEGF level and the number of neutrophils of various subpopulations can be due to the fact that in the absence of clinically significant ischemic syndromes (patients with ASCVD were not included in the study), the serum VEGF level does not represent local tissue hypoxia, which was previously shown in examples of other diseases [29, 30].

The presented study has some limitations: (1) the absence of a noninvasive assessment of plaque neovascularization in vivo, which would confirm its mediating role in the extent lesion of the carotid arteries with a decrease in circulating proangiogenic neutrophils; (2) the immunohistochemical examination of AP ex vivo with the determination of the content of VEGFR2^hi^-neutrophils which could confirm the accumulation of this subtype of neutrophils in AP with a decrease in their number in the systemic circulation; and (3) a limited sample size. One of the main results of our study was to establish the diagnostic efficacy of VEGFR2^hi^-neutrophils in detecting AP with a maximum height > 2.1 mm by ROC analysis. Using MedCalc software, we calculated a sufficient sample size to perform a valid ROC analysis, taking into account the received AUC values and the ratio of positive/negative cases. The required total number of patients was 153 (actual number 145); the number of positive cases was 21 (actual number 20). Thus, there is a minor underachievement in the optimal number of patients, mostly from the group of negative cases. However, in our opinion, our findings are important in view of the hypothesis-generating type of study.

## 5. Conclusions

In patients aged 40-64 years without established ASCVD, with an increase in indicators of the carotid plaque burden, a significant decrease in the proportion of circulating VEGFR2^hi^-neutrophils was noticed. A decrease in the relative number of VEGFR2^hi^-neutrophils of less than 4.52% made it possible to predict the presence of extent carotid atherosclerosis with sensitivity of 78.9% and specificity of 61.5%.

## Figures and Tables

**Table 1:** Clinical and laboratory characteristics of patients.

Characteristics	Patients (*n* = 145)
Male, *n* (%)/female, *n* (%)	75 (51.7)/70 (48.3)
Age (years), Ме (LQ; UQ)	49.0 (44.0; 56.2)
BMI (kg/m^2^), Ме (LQ; UQ)	26.9 (24.0; 30.4)
Obesity, *n* (%)	37.0 (25.5)
Abdominal obesity, *n* (%)	79 (54.5)
Smoking, *n* (%)	28 (19.3)
T2DM, *n* (%)	8 (5.51)
Hypertension, *n* (%)	77 (53.1)
Dyslipidemia, *n* (%)	129 (88.9)
*Β*eta-blockers, *n* (%)	29 (20.0)
Renin-angiotensin system inhibitors, *n* (%)	38 (26.2)
Diuretics, *n* (%)	13 (8.96)
Statins, *n* (%)	38 (26.2)
Leukocytes (cells × 10^9^/l), Ме (LQ; UQ)	5.75 (4.70; 6.90)
TC (mmol/l), Ме (LQ; UQ)	5.81 (5.03; 6.62)
LDL-C (mmol/l), Ме (LQ; UQ)	3.53 (3.00; 4.40)
HDL-C (mmol/l), Ме (LQ; UQ)	1.36 (1.19; 1.60)
TG (mmol/l), Ме (LQ; UQ)	1.30 (0.90; 1.78)
hsCRP (mg/l), Ме (LQ; UQ)	2.51 (1.25; 3.12)
VEGF (pg/ml), Ме (LQ; UQ)	309.4 (220.3; 401.6)
Glycated hemoglobin (%), Ме (LQ; UQ)	5.61 (5.16; 5.99)
eGFR (ml/min/1.73 m^2^), Ме (LQ; UQ)	70.0 (61.0; 85.5)
cIMTm (mm), Ме (LQ; UQ)	0.645 (0.575; 0.710)
fIMTm (mm), Ме (LQ; UQ)	0.650 (0.550; 0.716)
Carotid plaque, *n* (%)	98 (67.6)
Maximal carotid stenosis (%), Ме (LQ; UQ)	25.0 (0.00; 30.0)
cPTm (mm), Ме (LQ; UQ)	1.70 (1.40; 2.10)

BMI = body mass index; TC = total cholesterol; HDL-C = high-density lipoprotein cholesterol; LDL-C = low-density lipoprotein cholesterol; TG = triglycerides; eGFR = estimated glomerular filtration rate; hsCRP = high-sensitivity C-reactive protein; T2DM = type 2 diabetes mellitus; VEGF = vascular endothelial growth factor; cIMTm = mean carotid intima-media thickness; fIMTm = mean femoral intima-media thickness; cPTm = maximal carotid plaque thickness.

**Table 2:** Results of flow cytofluorometry showing neutrophil subpopulation composition.

Cell type	Cell number	
	Absolute values (cells/*μ*l)	Relative values (%)
Neutrophils	3400 (2700; 4200)	59.0 (51.2; 66.0)
CD16^hi^CD11b^hi^CD62L^hi^ (mature neutrophils)	2851 (2309; 3531)	89.4 (82.5; 92.9)
CD16^hi^CD11b^br^CD62L^lo^CXCR4^hi^ (aging neutrophils)	268 (141; 478)	7.90 (4.34; 16.3)
CD16^hi^CD11b^hi^CD62L^lo^CXCR2^hi^VEGFR2^hi^ (proangiogenic neutrophils)	162 (86.0; 285)	5.00 (2.66; 8.12)

Cells/*μ*l = cells in 1 *μ*l.

## Data Availability

The data used to support the findings of this study are available from the corresponding author upon request.

## Supplementary Materials

Supplementary Materials The supplementary material file contains tables presenting data on the number of circulating neutrophils of different subpopulations depending on carotid plaque burden (Table S1) and linear regression analysis showing the effect of VEGFR2^hi^-neutrophils on cIMTm and fIMTm (Table S2).
