# Supplementary material for: Associations between Circulating VEGFR2hi-Neutrophils and Carotid Plaque Burden in Patients Aged 40-64 without Established Atherosclerotic Cardiovascular Disease
Source: J Immunol Res. 2022 Apr 26;2022:1539935. doi: 10.1155/2022/1539935 (PMC9064511; doi:10.1155/2022/1539935)
Supplement: Supplementary Materials — The supplementary material file contains tables presenting data on the number of circulating neutrophils of different subpopulations depending on carotid plaque burden (Table S1) and linear regression analysis showing the effect of VEGFR2hi-neutrophils on cIMTm and fIMTm (Table S2). [file 1539935.f1.docx]

**Supplementary Material**

Table S1. Neutrophils of different subpopulations in relation to the carotid plaque burden.

Table S2. Linear regression analysis showing the effect of VEGFR2^hi^-neutrophils on cIMTm and fIMTm

Table S1. Neutrophils of different subpopulations in relation to the carotid plaque burden

| Cells | Q1 | | Q2 | | Q3 | | Q4 | | p | |
| --- | --- | --- | --- | --- | --- | --- | --- | --- | --- | --- |
|  | cells/μl | % | cells/μl | % | cells/μl | % | cells/μl | % | cells/μl | % |
| CD16^hi^CD11b^hi^CD62L^hi^ (mature neutrophils) | 2915 (2338; 3697) | 90.0 (82.4; 92.6) | 2638 (2325; 2986) | 87.4 (80.2; 92.4) | 3016 (2084; 4380) | 89.8 (87.2; 93.7) | 3045 (2463; 3238) | 88.5 (83.3; 91.1) | 0.499 | 0.411 |
| CD16^hi^CD11b^br^CD62L^lo^CXCR4^hi^ (aging neutrophils) | 278 (149; 580) | 8.00 (4.16; 18.5) | 246 (125; 396) | 7.58 (4.59; 12.8) | 288 (186; 499) | 9.13 (5.19; 16.1) | 252 (155; 411) | 7.13 (3.96; 10.8) | 0.669 | 0.652 |
| CD16^hi^CD11b^hi^CD62L^lo^CXCR2^hi^ VEGFR2^hi^ (proangiogenic neutrophils) | 164 (91.0; 281) | 4.84 (2.69; 8.11) | 147 (75.0; 280) | 5.17 (2.89; 9.50) | 209 (106; 336) | 6.66 (3.40; 8.84) | 94 (64.0; 187) | 3.08 (2.04; 4.91) | 0.058 | 0.036 |

*The data in the table is presented as Me (LQ; UQ). The following quartiles (Q) are defined for the cPTm values: Q1 = <1.40 mm; Q2 = 1.40-1.70 mm; Q3 = 1.71-2.10 mm; Q4 = >2.10 mm.*

Table S2. Linear regression analysis showing the effect of VEGFR2^hi^-neutrophils on cIMTm and fIMTm

| Characteristics | R | R^2^ | B | 95% CI for B | | p |
| --- | --- | --- | --- | --- | --- | --- |
|  |  |  |  | Lower limit | Upper limit |  |
| cIMTm | | | | | | |
| VEGFR2^hi^-neutrophils, % | 0.281 | 0.079 | -0.005 | -0.008 | -0.002 | 0.001 |
| fIMTm | | | | | | |
| VEGFR2^hi^-neutrophils, % | 0.233 | 0.054 | -0.005 | -0.008 | -0.001 | 0.006 |

*cIMTm = mean carotid intima-media thickness; fIMTm = mean femoral intima-media thickness*
